# Platelet Activation Is Limited during Transcatheter Aortic Valve Implantation in Patients on Aspirin Monotherapy and without per Procedural Clinical Complications

**DOI:** 10.1055/s-0039-1692142

**Published:** 2019-05-30

**Authors:** Nicolas Dumonteil, Marie Levade, Cédric Garcia, Thibault Lhermusier, Jennifer Series, Pauline Le Faouder, Bertrand Marcheix, Bernard Payrastre, Didier Carrié, Pierre Sié

**Affiliations:** 1 Pôle Cardiovasculaire et Métabolique, Hôpital Rangueil, Toulouse, France; 2 INSERM, U1048 and Université Toulouse 3, Institut des Maladies Métaboliques et Cardiovasculaires, Toulouse, France; 3 Laboratoire d'Hématologie, Centre Hospitalier Universitaire de Toulouse, Toulouse, France; 4 MetaToul-Lipidomique, INSERM U1048 and Université Paul Sabatier Toulouse 3, Toulouse, France

**Keywords:** platelet activation, aortic valve stenosis, transcatheter aortic valve implantation

## Abstract

Transcatheter aortic valve implantation (TAVI) is an established treatment option for symptomatic patients with severe aortic valve stenosis (AS). During and early after the procedure, both ischemic events (predominantly stroke) and bleedings remain prevalent. The optimal antithrombotic regimen is still debated. Single- versus dual-antiplatelet therapy is associated with a lower rate of severe bleeding, without difference in thrombotic complications. Although platelets have been empirically targeted, little is known on their contribution to these events primarily related to embolization of thrombotic material and tissue-derived debris from the wounded aortic valve and large vessels. The objective of this study was to assess local platelet activation in blood sampled in the ascending aorta immediately before and within minutes postimplantation. A series of 18 patients with AS on monotherapy with aspirin successfully underwent TAVI with the self-expandable Medtronic CoreValve by transfemoral route. No clinical thrombotic complication occurred at 30-day follow-up. Compared with patients with stable coronary artery disease unscathed of AS and similarly treated by low-dose aspirin, AS patients displayed a chronic state of platelet activation before TAVI, assessed in venous blood using various biomarkers. However, per procedure, in aortic blood, no change occurred between the two time points in the plasma levels of serotonin or 12-lipoxgenase products, or membrane exposure of granule markers CD62-P and CD63. Our results suggest that local acute platelet activation is limited during TAVI on monotherapy with aspirin.

## Introduction

Transcatheter aortic valve implantation (TAVI) is currently the standard of care for the treatment of symptomatic severe aortic valve stenosis (AS) for patients with high surgical risk. 1 In spite of improvement of devices and operators' experience and better patient assessment during the last decade, TAVI still carries a significant periprocedural thromboembolic and concomitant bleeding risk 2 that fuels the debate on the optimal antithrombotic therapy in patients undergoing TAVI. 3 4 Although clinically apparent cerebral ischemia and persistent neurological impairment rarely occur, the incidence of clinically silent cerebral embolic lesions after TAVI is high. Most strokes occur in the acute phase (first day) and are strongly related to procedural factors. 3 Transcranial Doppler has demonstrated the role of interaction of the device with the native aortic valve as the main cause of cerebral embolization. 5 Captured debris in filters deployed in large cerebral arteries during the procedure associate thrombotic material to tissue-derived debris. 6 However, the mechanical stress to the aorta and to the calcified native valve caused by catheter manipulation, balloon dilation, retrograde valve positioning, and frame expansion induces a high thrombogenic surface that could strongly activate the platelets and the coagulation pathways until endothelialization. Although the contribution of platelets to acute thromboembolic events may be a potential important issue, it has never been directly investigated. To investigate this point, we have measured local platelet activation in blood sampled in the ascending aorta immediately before and within minutes postimplantation before removing the catheter, in patients undergoing TAVI. Platelet activation in aortic whole blood was assessed by measurement of secretion of serotonin and bioactive lipids and expression of membrane granule markers and compared between the two time points. All patients were on monotherapy with aspirin according to our local protocol based on a low rationale for dual-antiplatelet pretreatment before TAVI. 7 The primary laboratory endpoint of the study was the comparison of platelet activation markers in aortic whole blood sampled pre- and immediately post-TAVI. The secondary laboratory endpoint was the comparison of platelet activation markers in peripheral venous blood before TAVI between patients with severe aortic stenosis (AS group) and patients with stable coronary artery disease (CAD) without aortic stenosis (CAD group).

## Methods

### Patients

This prospective, monocentric, observational study (NCT02504632) was performed in 20 high-risk patients with severe symptomatic AS (aortic valve area [AVA] <1 cm ^2^ or 0.6 cm ^2^ /m ^2^ of body surface area) undergoing TAVI for indications according to current recommendations. 8 9 Patients were not included if they had acute coronary syndrome 1 month before inclusion, terminal chronic kidney disease requiring hemodialysis, baseline indication of dual-antiplatelet therapy (DAPT) or current anticoagulant treatment, thrombocytopenia less than 100 G/L, or hemoglobin less than 100 g/L. The patients received a monotherapy by aspirin (75–160 mg/d) for at least 1 week before TAVI.

A group of 26 patients admitted for coronary angiogram assessment for stable CAD served as comparator. These patients were unscathed of AS, based on cardiac echography performed mainly as outpatients in the months before, and on clinical exam at admission.

The research protocol complies with the Declaration of Helsinki and was approved by the Toulouse University Hospital Human Research and Ethics Committee. Informed consent was obtained from all participants.

### Transcatheter Aortic Valve Implantation Procedure

The third-generation Medtronic CoreValve ReValving System (Medtronic, Minneapolis, Minnesota, United States) was implanted by the same operator in all patients by transfemoral approach with double Pro-Glide (Abbott Vascular, Redwood City, California, United States) preclosing, as previously published. 10 Procedures were performed under anticoagulation by a bolus of unfractionated heparin at weight-adjusted dose.

The clinical endpoints after TAVI were described following Valve Academic Research Consortium-2 definitions. 11 In-hospital clinical, biological, and transthoracic echocardiography (TTE) follow-up was performed before discharge. Active 30-day follow-up was obtained in all survivors by outpatient visit or direct contact with their cardiologist. All events and values were prospectively site recorded.

### Blood Collection and Preparation of Samples

Aortic blood was sampled into 0.109 M sodium citrate through the pig-tail catheter downstream aortic valve, just before (T1) and 10 to 15 minutes after (T2) the implantation of the prosthesis. A venous blood sample was collected by forearm venepuncture at the beginning of the procedure, before heparin administration, and manipulation of the aortic valve. Platelet-rich plasma (PRP) and platelet-poor plasma (PPP) were immediately obtained by differential centrifugation. PPP for measurement of soluble markers and PPP mixed with cold methanol (1:1, v:v) for eicosanoid quantification were stored at −80°C, whereas platelet membrane markers of granule secretion were assessed on fresh PRP.

### Laboratory Methods

Commercial enzyme-linked immunosorbent assays were used for quantification of soluble markers such as soluble platelet glycoprotein VI (sGPVI; MyBioSource, San Diego, California, United States), sP-selectin, and sCD40-ligand (eBioscience, Paris, France) and serotonin (IBL-America, Minneapolis, Minnesota, United States) in PPP. Platelet aggregation in response to 5 µM adenosine diphosphate (ADP, Sigma), 50 µM thrombin receptor activating peptide (TRAP, Sigma), or 10 µg/mL collagen-related peptide (CRP, from Prof. Farndale, University of Cambridge, UK) was studied in PRP using light transmission aggregometry (TA-8V aggregometer, SD Medical). Results are expressed in % maximal aggregation (reference laboratory values: 70–100%). Platelet granule secretion was assessed by measuring transmembrane glycoproteins CD62-P and CD63, markers of α and δ granules, respectively, by flow cytometry. PRP was incubated with anti-CD62P FITC or anti-CD63 FITC-conjugated antibodies (BD Pharmingen, BD Biosciences, Le Pont-de-Claix, France) for 15 minutes at room temperature in the dark. The analysis was performed using a BD FACS Verse cytometer (BD Biosciences). Results were expressed as median fluorescence intensity (MFI) for unstimulated platelets (upper normal limit: 40 arbitrary units), or as MFI-fold increase over resting value in PRP stimulated with 10 µg/mL CRP or 50 µM TRAP (Sigma-Aldrich, Saint-Quentin-Fallavier, France) for 10 minutes at 37°C.

Plasma eicosanoids were quantified by liquid chromatography-tandem mass spectrometry (LC-MS/MS) as previously described. 12 Internal standard containing LxA4-d5, LtB4-d4, and 5-HETE-d8 at 400 ng/mL (Cayman Chemicals, Ann Arbor, Michigan, United States) was added to the plasma/methanol before lipid extraction. Lipoxins A4 and B4; resolvins D1, D2, and 7(S)-maresin; leukotrienes B5, 10(S), and 17(S)-protectin; 9- and 13-hydroxyoctadecadienoic acids; 5-, 12-, and 15-hydroxyeicosatetraenoic acids (HETE); and 14- and 17-hydroxydocosahexaenoic acid (HDoHE) were separated on a ZorBAX SB-C18 column using Agilent 1290 Infinity HPLC system coupled to an ESI-triple quadruple G6460 mass spectrometer (Agilent Technologies, Santa Clara, California, United States). Data were acquired in Multiple Reaction Monitoring mode with optimized conditions (ion optics and collision energy). Peak detection, integration, and quantitative analysis were done using Mass Hunter Quantitative analysis software (Agilent Technologies) based on calibration lines built with internal standards.

### Study Endpoints

The clinical endpoints after TAVI were described following Valve Academic Research Consortium definitions. 11 In-hospital clinical, biological, and TTE follow-up was performed before discharge. Thirty-day follow-up was active and was obtained in all survivors by medical visit or direct contact with their cardiologist. All events and values were prospectively site recorded.

The primary laboratory endpoint was the comparison of platelet activation markers in aortic whole blood sampled pre- and immediately post-TAVI. The secondary laboratory endpoints were the comparison before TAVI of platelet activation markers in peripheral venous blood with the values measured in aortic blood at T1 and with venous blood of CAD patients.

### Statistical Analysis

Sample size was calculated according to the primary objective of the study, that is, to assess local platelet activation in aortic blood within minutes following prosthetic valve implantation (T2).

In static conditions, in vitro platelet secretion induced by soluble agonists produces a large increase of cytokines or bioactive lipids released in plasma. We assumed that, if ∼10% of circulating platelets were activated by passing through the valve, the levels of plasma markers will double in the aortic blood sampled close to the injured vessel. Given this assumption and the standard deviations provided for the various hemostasis parameters in preliminary analyses, we calculated that 20 subjects would be needed to assess such a doubling of hemostasis parameters between T1 (before the procedure) and T2, under the hypothesis of a 90% power, a 5% type 1 error rate, and using a paired nonparametric test.

Statistical analysis was performed using GraphPad Prism software. Continuous variables were expressed as mean ± standard deviation, or median (interquartile range [IQ]: 25th–75th) as appropriate, and compared between groups or between time points using the nonparametric Mann–Whitney or paired Wilcoxon's tests. Multilinear regression analysis with age and group was performed on matched population. Discrete variables were expressed as number and percentage, and compared using the Fisher test.

## Results

### Baseline Characteristics of Patients and Procedural Clinical Outcomes

The results of two included patients with AS were removed for technical reasons. The baseline characteristics of the other 18 patients with AS are depicted in Table 1 . None of them had bleeding symptoms before the procedure. The success rate of VARC-2 device was 100%. Within the first 24 hours, platelet counts significantly dropped by 23% from 214 ± 71 to 165 ± 51 G/L, and hemoglobin by 12% from 121 ± 15 to 107 ± 17 g/L, whereas a transient increase in leukocyte count occurred, from 7.4 ± 2.1 G/L to 9.4 ± 2.6 G/L. One death related to a cardiac tamponade occurred few hours after the procedure. The other 17 patients reached the 30-day follow-up visit alive, with a functional improvement and a good function of the aortic bioprosthesis (mean AVA: 1.9 ± 0.4 cm ^2^ , 16 patients with none or trace and 1 with mild paravalvular regurgitation on TTE).

**Table 1:** Key baseline demographic and clinical data of patients in AS before TAVI and CAD groups

	AS ( *n* = 18)	CAD ( *n* = 26)	*p*
Age (y), mean ± SD	86 ± 4	68 ± 12	< 10 ^−4^
Male, *n* (%)	10 (55)	19 (73)	0.53
Body mass index, mean ± SD	25 ± 4	27 ± 6	0.23
New York Hearth Association class III or IV, *n* (%)	15 (83)	0	< 10 ^−4^
Logistic EuroSCORE, mean ± SD	21 ± 11	NA	
Coronary artery disease, *n* (%)	12 (66)	24 (92)	0.048
Diabetes mellitus, *n* (%)	4 (22)	9 (34)	0.34
Hypertension, *n* (%)	12 (66)	18 (69)	1.0
Chronic obstructive pulmonary disease, *n* (%)	3 (16)	1 (4)	0.29
Peripheral vascular disease, *n* (%)	0	6 (23)	0.07
Creatinine (µmol), mean ± SD	125 ± 53	93 ± 22	0.011
Hemoglobin (g/L), mean ± SD	121 ± 15	141 ± 13	< 10 ^−3^
Platelet count (G/L), mean ± SD	214 ± 71	254 ± 70	0.081
Echocardiography, mean ± SD			
Aortic valve area (cm ^2^ )	0.77 ± 0.11	NA	
Left ventricular ejection fraction (%)	51.7 ± 11.9	NA	
Mean pressure gradient (mm Hg)	43.3 ± 14.5	NA	

Abbreviations: AS, aortic valve stenosis; CAD, coronary artery disease; NA, not available; SD, standard deviation; TAVI, transcatheter aortic valve implantation.

Note: Continuous variables were compared using the nonparametric Mann–Whitney test and discrete variables using Fisher's test.

The group of 26 patients with stable CAD were significantly younger, have no cardiac failure above NYA class II, and by definition most of them had CAD confirmed on angiography ( Table 1 ). Creatinine and hemoglobin levels were, respectively, lower and higher in the CAD group than in the AS group.

### Platelet Activation Markers in Venous Blood before TAVI Procedure

Compared with patients with stable CAD, the plasma levels of biomarkers of platelet activation, sGPVI and sCD40L, shed from the platelet membrane, and products of platelet lipid metabolism, were significantly higher in the AS group. However, after multivariate regression analysis, using age and groups as covariable, the difference was nonsignificant for sGPVI and sCD40L. Among the different bioactive lipids analyzed, we focused on the main products of 12-lipoxygenase (LOX), 12-HETE, and 14-HDoHE, released in plasma upon platelet activation. These two lipids were significantly correlated ( *r* ^2^ = 0.66, *p* = 0.007). As our patients were taking aspirin, cyclooxygenase (COX) products, especially thromboxane B2, were undetectable. The levels of other detectable lipid products on the LC-MS/MS profile (5-HETE, 15-HETE, 9-HODE, and 13-HODE), mainly produced by leukocytes and endothelial cells, were not significantly different between the two patient groups (not shown). Plasma levels 12-HETE and 14-HDoHE remained significantly higher in AS group compared with CAD after adjustment for age ( *p* = 0.003 and 0.007, respectively). Membrane exposure of CD62-P and CD63 by unstimulated platelets was low in both patient groups, with MFI in the normal reference range of the laboratory. Platelet aggregation (maximal amplitude) induced by ADP, CRP, and TRAP in PRP was in the low normal range in both groups, as usually found under aspirin therapy. Only CRP-induced aggregation was slightly, but significantly, lower in AS patients, possibly due to loss of membrane GPVI. Membrane exposure of CD62P and CD63 was induced by CRP or TRAP at similar extent in both groups. These results suggest that platelets circulating in peripheral blood are not desensitized by multiple passages through the stenotic valve and that, in static conditions, circulating platelets were reactive to soluble agonists ( Table 2 ).

**Table 2:** Baseline platelet activation markers and in vitro platelet reactivity in AS before TAVI and control groups

Platelet activation markers in venous blood		AS ( *n* = 18)	CAD ( *n* = 26)	*p*
Soluble CD40 ligand (ng/mL), median [IQ ^25–75^ ]		3.3 ^[0.9–6.6]^	1.0 ^[0.6–1.4]^	0.027
Soluble P-selectin (ng/mL), median [IQ ^25–75^ ]		69 ^[56–97]^	61 ^[48–86]^	0.30
Soluble GPVI (ng/mL), median [IQ ^25–75^ ]		58 ^[26–80]^	16 ^[5–44]^	0.026
12-HETE (ng/mL) median [IQ ^25–75^ ]		9.0 ^[7–21]^	2.6 ^[1.5–3.9]^	<10 ^3^
14-HDoHE (ng/mL) median [IQ ^25–75^ ]		2.7 ^[1.9–7.3]^	0.7 ^[0.4–0.9]^	<10 ^3^
Maximal amplitude of platelet aggregation in PRP, mean ± SD	ADP 5 µM CRP 10 µg/mL TRAP 50 µM	58 ± 16 66 ± 11 71 ± 11	58 ± 19 80 ± 10 75 ± 9	0.8 <0.01 0.5
CD62-P surface exposure (MFI) on resting platelets, median [IQ ^25–75^ ]		35 ^[12–59]^	25 ^[9–46]^	0.25
CD63 surface exposure (MFI) on resting platelets, median [IQ ^25–75^ ]		26 ^[20–28]^	23 ^[20–54]^	0.70
CD62-P MFI fold-increase on stimulated platelets in PRP vs. resting platelets, median [IQ ^25–75^ ]	CRP 10 µg/mL	23 ^[12–38]^	26 ^[15–49]^	0.56
	TRAP 50 µM	4 ^[2–12]^	16 ^[4–23]^	0.07
CD63 MFI fold-increase on stimulated platelets in PRP vs. resting platelets	CRP 10 µg/mL	14 ^[8–19]^	18 ^[10–22]^	0.20
	TRAP 50 µM	4 ^[2–8]^	7 ^[2–13]^	0.48

Abbreviations: ADP, adenosine diphosphate; AS, aortic valve stenosis; CAD, coronary artery disease; CRP, collagen-related peptide; MFI, median fluorescence intensity; PRP, platelet-rich plasma; SD, standard deviation; TAVI, transcatheter aortic valve implantation; TRAP, thrombin receptor activating peptide.

Note: The results of the two groups were compared using the non-parametric Mann–Whitney test.

### Local Platelet Activation in Aortic Blood during TAVI Procedure

To catch platelet activation on the surface of injured valve, we measured platelet activation in blood sampled in the ascending aorta immediately before (T1) and within minutes postimplantation, before removing the catheter (T2). At T1, the levels of bioactive lipids (13.7 ^[6.0–32.2]^ ng/mL for 12-HETE and 3.5 ^[1.9–7.5]^ ng/mL for 14-HDoHE, median ^[IQ 25–75]^ ) were nonsignificantly different from those measured in venous blood at the same time (see Table 2 , *p* = 0.74 and 0.38, respectively, paired Wilcoxon's test). Similarly, membrane exposure of CD62-P or CD63 by resting platelets was low. This rules out platelet activation induced by blood collection through the transfemoral catheter or induced by unfractionated heparin at therapeutic concentrations administered before the procedure. 13

**Fig. 1:**
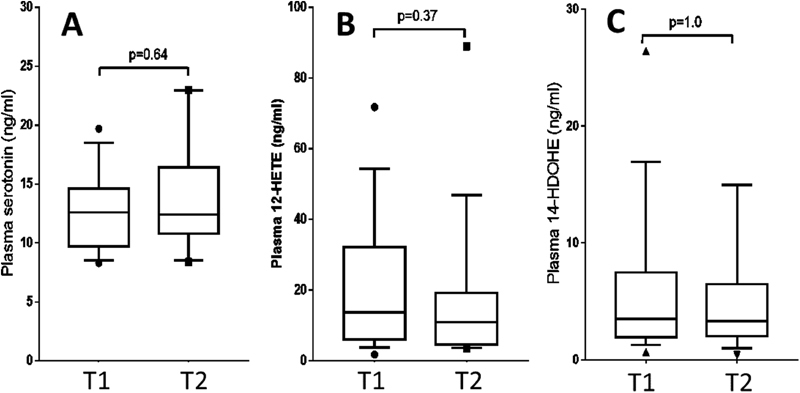
Platelet activation markers in aortic blood sampled immediately before (T1) and 10–15 minutes (T2) after the placement of the aortic bio prosthesis. Whisker boxes: 10–25–75–90th percentiles and outliers. Statistical significance calculated using nonparametric paired Wilcoxon's test.

As shown in Fig. 1A–C , 12-HETE, 14-HDoHETE, and serotonin demonstrated no significant change between the two time points. As expected for two products of the same metabolic pathway, 12-HETE and 14-HDoHE levels were linearly correlated, both at T1 ( *r* ^2^ = 0.40, *p* < 10 ^3^ ) and T2 ( *r* ^2^ = 0.59, *p* < 10 ^3^ ). In contrast, they were not correlated with serotonin levels at any time ( *p* = 0.89 and 0.93, respectively). Membrane exposure of CD62-P (MFI ^[IQ 25–75]^ : 33 ^[16–57]^ and 38 ^[11–67]^ before and after, respectively, *p* = 0.67), or CD63 (27 ^[23–32]^ and 30 ^[17–32]^ , *p* = 0.36) did not vary and remained low.

## Discussion

The association between shear stress and hemostatic activation has been demonstrated in vitro and in vivo in various clinical conditions, severe aortic stenosis being one of the best documented. Increased levels of sCD40L or sGPVI 14 15 in conjunction with a reduction of surface GPIba or GPVI, 16 platelet-derived chemokines 17 and serotonin 18 in platelet-free plasma, or of circulating platelet-derived microparticles 19 20 21 and platelet-monocyte aggregates 22 in whole blood have been reported in these patients. Platelet generation of eicosanoids resulting from the oxidative transformation of arachidonic acid through cyclooxygenase 1 (COX-1) and lipoxygenase (LOX) pathways is an early response to platelet activation. 23 The development of lipidomics for sensitive and accurate quantitation of a large set of eicosanoids introduces potential new biomarkers of platelet activation, investigated for the first time in AS in our study. As all patients in both groups received low-dose aspirin, COX-1 products were not measurable and the flux of arachidonic acid was redirected to LOXs, mainly 12-LOX, the most abundant in platelets. 23 The two main products released in plasma were 12-HETE and 14-HDoHE. The variability of plasma 12-HETE in our cohort was relatively large, as reported in collagen-stimulated platelets of healthy volunteers before or after aspirin administration. 24 Although low-dose aspirin has also been reported to affect 12-LOX and to partially reduce 12-HETE production by collagen-stimulated platelets, 24 the higher levels in patients with AS compared with those with CAD similarly treated by aspirin ( Table 2 ), which remains after adjustment for age, are indicative of in vivo platelet activation induced by the pathological shear stress.

Compared with peripheral venous blood, the eicosanoid levels in aortic blood before TAVI were not significantly different and platelets did not expose more CD62-P or CD63. This suggests that the subset of platelets activated by high shear through the valve is too small for being detected downstream by flow cytometry. As activated platelets are rapidly removed from the circulation or do not circulate as isolated cells, they cannot be detected either in peripheral blood, in contrast to soluble biomarkers that persist longer. In addition, systemic activation by various factors besides high shear stress, such as left ventricular dysfunction, advanced atherosclerosis, or low-grade inflammation, may be preeminent.

The main result of the study is the absence of detectable platelet activation per procedure in blood sampled close to the injured valve ( Fig. 1 ). Captured debris in filters deployed in large cerebral arteries during TAVI associate thrombotic material to tissue-derived debris dislodged from the degenerated valve or the aorta. 4 Activated platelets associated with such mixed material would escape to flow cytometry analysis. However, an increase of soluble markers serotonin and lipoxins was expected in blood collected close to the site of platelet interactions with the crushed aortic valve, before their dilution in systemic circulation. Although we showed normal reactivity of circulating platelets to soluble agonists in static conditions ( Table 2 ), the shedding of platelet membrane receptors GPIbα and GPVI induced by high shear, 16 25 combined with the defect of high-molecular-weight von Willebrand factor, 26 27 28 low-dose aspirin treatment, and thrombin inhibition by heparin, may explain our unexpected negative results.

This study has several limitations: (1) The series is short. However, there is no trend of acute change before and immediately after the procedure close to the aortic valve. (2) Only one time point after implantation is available. This was constrained by technical/safety reasons. However, it is unlikely that the bioprosthesis would provide a complete coverage of the thrombogenic surface within minutes after implantation. (3) The sensitivity and specificity of the biomarkers may be insufficient. However, the convergent results obtained with secreted, synthesized, and surface-bound markers confirm a chronic platelet activation reported by others in patients with severe AS, but they do not suggest acute per procedure activation. (4) Only one model of self-expandable bioprosthesis was used in this study. Platelet activation dependent on the model has been reported, associated with residual aortic regurgitation. 29 In our study, only 1/17 patient had mild paravalvular leakage on TEE at discharge. (5) Finally, and most importantly, no patient developed a clinically apparent ischemic event after valve implantation. Our results do not exclude a contribution of platelets to such complication when it occurs.

Patients undergoing TAVI are at high risk for both ischemic stroke and bleeding, most of them occurring early after the procedure, but the risk of severe bleeding is approximately twice that of ischemic complications. A recent meta-analysis comparing monotherapy with aspirin (SAPT) to DAPT indicated that DATP was associated with a higher rate of major adverse events after TAVI, mainly driven by an increased risk of major or life-threatening bleeding complications along with a lack of beneficial effect on ischemic events. 30 In patients treated with DATP, low (vs. high) on-treatment platelet reactivity is associated with more bleeding events without difference in ischemic events at 30-day follow-up. 31 In a recent study, no difference in the rates of ischemic complications was observed between patients who received a loading dose of clopidogrel, compared with those who did not. 32 Although we did not compare patients under DAPT vs. SAPT, our results are consistent with these findings. They suggest that during uneventful TAVI on monotherapy with aspirin, acute per procedure platelet activation remains limited. However, due to the small sample size of the population, this analysis is a hypothesis-generating case series and thus the conclusions should be confirmed in a larger study.
